# Impairment of adult hippocampal neural progenitor proliferation by methamphetamine: role for nitrotyrosination

**DOI:** 10.1186/1756-6606-4-28

**Published:** 2011-06-27

**Authors:** Arun Venkatesan, Lerna Uzasci, Zhaohui Chen, Labchan Rajbhandari, Carol Anderson, Myoung-Hwa Lee, Mario A Bianchet, Robert Cotter, Hongjun Song, Avindra Nath

**Affiliations:** 1 Department of Neurology, Johns Hopkins University School of Medicine, 600 N. Wolfe St., Baltimore, MD 21287, USA; 2 Middle Atlantic Mass Spectrometry Laboratory, Johns Hopkins University School of Medicine, 600 N. Wolfe St., Baltimore, MD 21287, USA; 3 Pharmacology and Molecular Sciences, Johns Hopkins University School of Medicine, 600 N. Wolfe St., Baltimore, MD 21287, USA; 4 Biophysics and Biophysical Chemistry, Johns Hopkins University School of Medicine, 600 N. Wolfe St., Baltimore, MD 21287, USA; 5 Institute for Cell Engineering, Johns Hopkins University School of Medicine, 600 N. Wolfe St., Baltimore, MD 21287, USA; 6 The Solomon H. Snyder Department of Neuroscience, Johns Hopkins University School of Medicine, 600 N. Wolfe St., Baltimore, MD 21287, USA; 7 National Institutes of Health, Section of Infections of the Nervous Systems, Bldg 10-CRC, Room 7C103; Bethesda, MD 20892

## Abstract

Methamphetamine (METH) abuse has reached epidemic proportions, and it has become increasingly recognized that abusers suffer from a wide range of neurocognitive deficits. Much previous work has focused on the deleterious effects of METH on mature neurons, but little is known about the effects of METH on neural progenitor cells (NPCs). It is now well established that new neurons are continuously generated from NPCs in the adult hippocampus, and accumulating evidence suggests important roles for these neurons in hippocampal-dependent cognitive functions. In a rat hippocampal NPC culture system, we find that METH results in a dose-dependent reduction of NPC proliferation, and higher concentrations of METH impair NPC survival. NPC differentiation, however, is not affected by METH, suggesting cell-stage specificity of the effects of METH. We demonstrate that the effects of METH on NPCs are, in part, mediated through oxidative and nitrosative stress. Further, we identify seventeen NPC proteins that are post-translationally modified via 3-nitrotyrosination in response to METH, using mass spectrometric approaches. One such protein was pyruvate kinase isoform M2 (PKM2), an important mediator of cellular energetics and proliferation. We identify sites of PKM2 that undergo nitrotyrosination, and demonstrate that nitration of the protein impairs its activity. Thus, METH abuse may result in impaired adult hippocampal neurogenesis, and effects on NPCs may be mediated by protein nitration. Our study has implications for the development of novel therapeutic approaches for METH-abusing individuals with neurologic dysfunction and may be applicable to other neurodegenerative diseases in which hippocampal neurogenesis is impaired.

## Background

Over 35 million people internationally abuse METH, and in the United States METH abuse has reached epidemic proportions. Through mechanisms that are not yet well understood, METH abusers suffer from a variety of neurocognitive deficits, including behavioral changes, executive dysfunction, deficits in perceptual speed and information manipulation, and impairment of verbal and spatial memory. Neurocognitive deficits may persist after cessation of METH abuse, are slow to improve, and may not completely reverse [1-5]. Although METH was initially thought to selectively damage monoaminergic nerve terminals, recent studies have consistently shown that widespread neuronal cell death results [6-10]. Cell death involves not only the striatum and cortex, but the hippocampus as well [7,11]. Although the molecular mechanisms underlying METH neurotoxicity are likely multifactorial, several key findings support a significant role for both oxidative and nitrosative stress. Mice overexpressing superoxide dismutase, an antioxidant, show markedly decreased METH-induced apoptosis [12]. Suppression of nitric oxide (NO) production, through both pharmacologic and genetic means, also protects against METH-mediated neurotoxicity. In addition, METH causes increased levels of 3-nitrotyrosine adduct formation, reflective of oxidative and nitrosative stress [13,14]. Although METH-induced oxidative and nitrosative stress and toxicity have been demonstrated in neurons, little is known about whether other cells within the brain, such as NPCs, are similarly affected by METH.

The brains of mammals contain several distinct populations of cells that are capable of dividing and differentiating into neurons and glial cells throughout adulthood [15,16]. Increasing evidence suggests that continued neurogenesis is important for maintenance of cognitive function [17-20]. Within the hippocampal dentate gyrus (DG), neurogenesis occurs in the subgranular zone and granule cell layer. New neurons formed here are functionally incorporated into the hippocampus [21] and may participate in the formation of hippocampal-dependent memory [22].

Studies in rodents have demonstrated that hippocampal neural progenitor cells (NPCs) can respond to a wide variety of environmental demands, such as enrichment and exercise, by increasing neurogenesis, with consequent enhancement in long-term potentiation of synaptic transmission as well as functional learning and memory [23-25]. On the other hand, neurogenesis is decreased in many settings in which learning and memory are disrupted in rodents, including aging, stress, inflammation, and exposure to certain drugs [16,26-29]. Of note, certain pathogenic conditions, such as epilepsy and stroke, lead to increased neurogenesis without a measurable improvement in cognition, possibly due to abnormal migration and integration of newly formed neurons [30]. Thus, pathogenic processes that either increase or decrease neurogenesis may interfere with cognitive function.

Although several drugs of abuse have been shown to influence neurogenesis [31,32], limited data are available with respect to the effects of METH. Several *in vivo *models of METH exposure have demonstrated decreases in hippocampal neurogenesis [33-35]. In addition, a recent study demonstrated that METH induces NPC death through mitochondrial fragmentation [36]. However, cellular and molecular mechanisms by which METH affects NPCs remain largely unknown. Here, we investigate mechanisms by which METH-induced oxidative and nitrosative stress impair NPC function.

## Results

### Characterization of cultures of adult hippocampal progenitor cells

**Figure 1:**
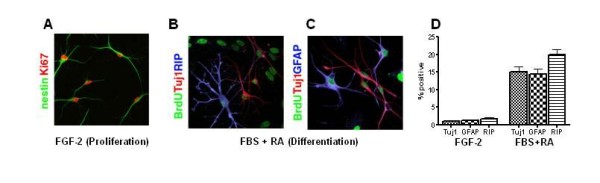
**Establishment of NPC culture system in vitro**. Adult hippocampal progenitors (NPCs) derived from Fisher rats were maintained under either proliferative or differentiating conditions. A. Under proliferative conditions, over 99% of cells are co-labeled with antibodies to nestin (green, cytoplasmic) and Ki67 (red, nuclear), markers of proliferating cells. B,C. Cells are exposed to proliferative conditions for 24 hrs in the presence of BrdU, followed by 5 days of differentiation conditions stain for BrdU (green), indicating that they were all initially proliferating cells. In addition, some cells in B co-label with Tuj1 (red, neuron), or RIP (blue, oligodendrocyte) and in C co-label with GFAP (blue, astrocyte). D. Immunostaining of cells derived from clonal NPCs grown under proliferative (FGF-2) or differentiating (FBS+RA) conditions yields reproducible percentages of Tuj1 (neuronal), GFAP (astrocytic), and RIP (oligodendroglial) positive cells.

NPCs were isolated from hippocampi of adult rats and maintained in culture media containing fibroblast growth factor (FGF) to ensure proliferation of these cells. Under these conditions, >99% of cells immunostained with antibody to nestin, a marker for NPCs (Figure 1A). These cells also stained for Ki67 a marker of proliferating cells (Figure 1A). To determine if these cells could be differentiated into various neural cell types, we initially treated them with BrdU to label the dividing cells and then changed the media to one containing fetal bovine serum (FBS) and retinoic acid (RA). Under these conditions, we found the presence of neuronal cells as demonstrated by immunostaining for Tuj1 (Figures 1B and 1C), astrocytes that immunostained for GFAP (Figure 1C) and oligodendrocytes that immunostained for RIP (Figure 1B). Nearly equal numbers (15-20%) of each of the cell types were present (Figure 1D). The remainder were undifferentiated NPCs. These self-renewing AHP thus fulfilled the definition of multipotent NPCs and were used for all further experiments.

### METH impairs proliferation and survival of NPCs

**Figure 2:**
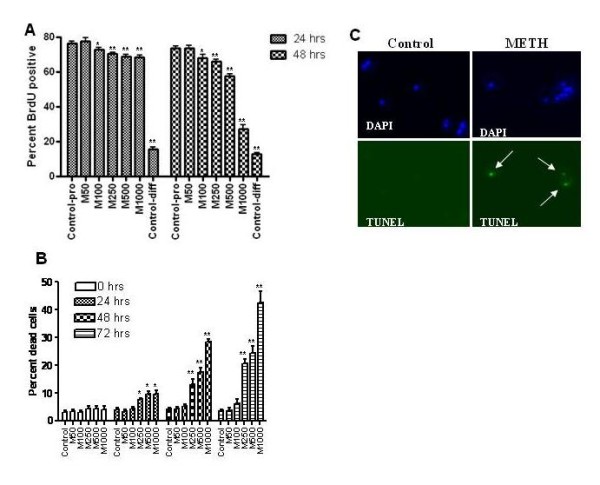
**METH decreases BrdU incorporation and causes apoptosis *in vitro***. NPCs maintained under proliferative conditions were exposed to various concentrations of METH for 48 hours. BrdU (5 uM) was added for 4 hours to label proliferating cells. Cells were immunostained with antibody against BrdU and counterstained with DAPI. A. Quantitation of BrdU positive cells reveals a dose- and time-dependent decrease in BrdU positive cells upon exposure to METH (numbers following METH refer to concentration in µM). * p <0.01, ** p <0.001 ANOVA compared to control (proliferation conditions). B. Cells were stained with Trypan Blue and visualized under light microscopy. Numbers following M refer to concentration of METH in µM. * p <0.01, ** p <0.001. C. TUNEL positive cells were not detected in control cells, but were seen in some cells exposed for 48 hrs to METH 1000 µM (white arrows).

To determine the effect of METH on proliferation of NPCs, these cells were exposed to METH for 24 or 48 hours, and then pulsed with BrdU for 4 hours. METH exposure resulted in a decrease in BrdU positive cells in both a dose- and time-dependent manner with significant effects at >100 uM METH (Figure 2A). After 48 hours at the highest concentration of METH used, only 25% of cells showed BrdU uptake.

We next considered that METH may directly cause NPC death, thus accounting for the reduction in proliferating NPCs. NPCs were exposed to METH for 24 to 72 hours, and cell death was quantified using several measures. Trypan blue staining demonstrated that METH caused a dose- and time-dependent increase in cell death (Figure 2B). Doses of >250 uM, resulted in significantly increased cell death. As opposed to BrdU uptake, dosages of 100 uM METH did not cause an increase in cell death even at 72 hours post treatment. At the highest concentration of METH, 50% cell death was seen at 72 hours (Figure 2B). Effects of METH on NPC death were also confirmed by 7-aminoactinomycin D (7-AAD) staining followed by flow cytometry. The amounts of dead cells determined by both techniques were similar (data not shown). To determine if METH could induce cell death via apoptosis, a TdT-mediated dUTP nick end-labeling (TUNEL) assay was performed. Exposure of NPCs to METH at doses of 250 uM, 500 uM (not shown) and 1 mM (Figure 2C) resulted in TUNEL positive cells, consistent with apoptotic DNA fragmentation.

### METH does not affect initial NPC differentiation

**Figure 3:**
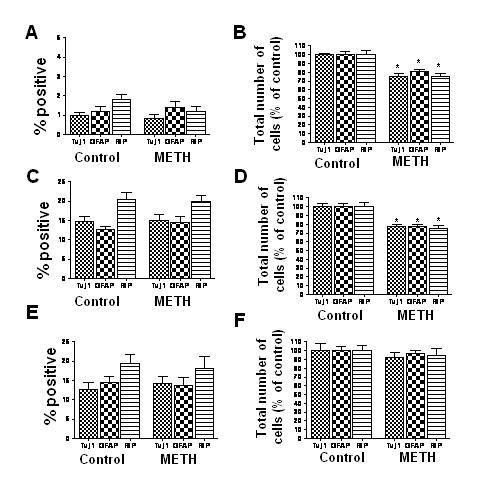
**METH does not affect differentiation of NPCs**. A,B. NPCs maintained under proliferative conditions were exposed to METH (250 uM) for 24 hours, and fixed and immunostained 4 days later with antibody against Tuj1, GFAP, and RIP, and counterstained with DAPI. METH treatment does not result in premature differentiation (A), but does result in fewer total numbers of differentiated cells (B). C,D. NPCs in proliferating media were exposed to METH for 24 hrs, followed by culture for 4 days in differentiating conditions. METH does not alter the percentage of cells adopting neuronal, astrocytic, or oligodendrocyte markers. E, F. NPCs were cultured in differentiating conditions for 24 hours, followed by METH treatment for an additional 24 hours. Cells were analyzed 4 days after being placed in differentiating conditions. METH treatment after the onset of differentiation does not significantly affect fate choice (E) or numbers of differentiating cells (F). * p <0.01 ANOVA, compared to control.

We considered the possibility that a reduction in proliferating NPCs upon exposure to METH may occur due to an increased drive toward differentiation. To investigate whether METH causes premature differentiation, we exposed proliferating NPCs to METH for 24 hours, and immunostained cells with markers of differentiating cells 4 days later. METH exposure did not result in premature differentiation of proliferating NPCs into neurons, astrocytes, or oligodendrocytes (Figure 3A), although total numbers of differentiated cells were reduced (Figure 3B). These results indicate that METH does not promote premature differentiation of proliferating NPCs. To further determine whether METH affects NPC differentiation, we treated proliferating NPCs with METH, followed by culture under differentiating conditions. Under these conditions, NPCs differentiated into neurons, astrocytes, and oligodendrocytes with the same frequency when exposed to METH as compared to control conditions (Figure 3C). Again, fewer differentiated cells were present in the METH-treated group as compared to controls (Figure 3D). Finally, we determined the effects of METH exposure on NPCs *after *the onset of differentiation. Under these conditions, too, METH did not significantly affect the frequencies of differentiating cells (Figure 3E). Interestingly, there was no reduction in total numbers of differentiated cells in the METH group as compared to control, suggesting that proliferating NPCs may be preferentially susceptible to the effects of METH as compared to differentiating NPCs.

### METH induces oxidative and nitrosative stress in NPCs

**Figure 4:**
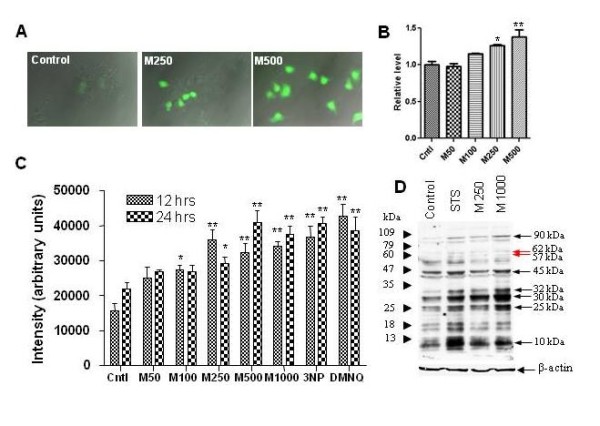
**METH induces oxidative stress in NPCs**. NPCs maintained under proliferative conditions were exposed to various concentrations of METH. Numbers following "M" refer to concentration of METH in uM. 3-NP, DMNQ, and STS are inducers of oxidative stress and were used as positive controls. A. NPCs exposed to METH for 4 hrs were loaded with Carboxy-H2 DCFDA. ROS formation (green) is far greater in cells exposed to METH (250 and 500 uM) than control. B. Nitrite production from supernatants of NPCs was assessed via the Griess reaction. Values were normalized to supernatants from untreated NPCs.C. 3-nitrotyrosination of cell lysates increases in a dose-dependent manner in NPCs exposed to METH as demonstrated by quantification of slot blots, suggesting increased oxidative and/or nitrosative stress. D. Lysates from untreated NPCs (Control) or those exposed to STS, METH 250 uM (M250) and 1000 uM (M1000) for 24 hours were subjected to western blotting with 3-nitrotyrosine antibody. Several bands, including those of apparent molecular mass 10, 25, 30, 32, 45, and 90 kDa, were preferentially nitrotyrosinated in NPCs exposed to METH (250 and 1000 uM) as compared to control cells. * p <0.05, ** p <0.01 ANOVA.

We determined whether the effects of METH on NPC proliferation and viability may be due to induction of oxidative and nitrosative stress. We first exposed NPCs to METH and measured intracellular reactive oxygen species using the fluorescent dye DCFDA. At concentrations of >100 uM, METH caused marked increases in DCFDA fluorescence at both 4 hours (Figure 4A) and 24 hours (not shown). To investigate nitrosative stress induced by METH, we assessed nitric oxide production, which has recently been shown to occur endogenously in hippocampal NPCs [37]. We employed the Griess reaction to measure nitrite formation, which occurs following the generation of nitric oxide. METH treatment resulted in nitrite formation in a dose-dependent fashion at 24 hrs (Figure 4B). To investigate the consequences of nitrosative and oxidative stress, we measured the formation of nitrotyrosine adducts on NPC proteins. 3-nitrotyrosination represents a common final pathway for the effects of both oxidative and nitrosative stress [38]. Semiquantitative analysis of 3-nitrotyrosine slot blots demonstrated that METH causes a dose-dependent increase in total 3-nitrotyrosination (Figure 4C). At concentrations of >250 uM METH, levels of nitrotyrosination were similar to those achieved by exposure to several potent inducers of oxidative stress. Western blotting analysis revealed that nitrotyrosination of several NPC proteins was increased in response to METH (Figure 4D). In particular, nitrotyrosination of proteins of approximate molecular masses 90 kDa, 45 kDa, 32 kDa, 30 kDa, and 25 kDa (black arrows) was increased by both METH and staurosporine (STS), an inducer of oxidative stress. Other proteins, such as those of approximate molecular mass 57 kDa (p57) and 62 kDa (p62) (red arrows), appeared not to be significantly nitrotyrosinated under control conditions, but were after METH exposure. Of note, differences in nitrotyrosination were found between STS- and METH-exposed NPCs; the protein of apparent molecular mass 30 kDa, for example, appears to be more strongly nitrotyrosinated upon METH exposure, while the protein of apparent mass 10 kDa appears more strongly nitrotyrosinated upon STS exposure.

**Figure 5:**
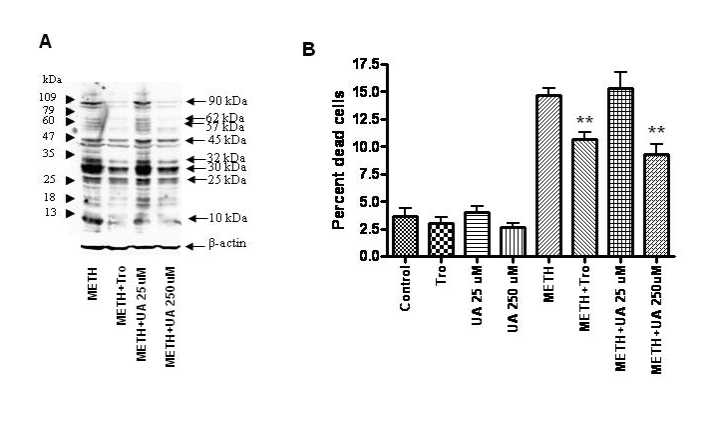
**Effects of antioxidants on METH-induced cell death of NPCs**. NPCs were preincubated for 2 hrs with either Trolox (10 uM) or uric acid (UA, 25 or 250 uM), followed by addition of METH (250 uM). Cells were analyzed at 48 hours. (A) Protein lysates were subjected to Western blotting with antibody against 3-nitrotyrosine. Both Trolox and Uric acid markedly decrease nitrotyrosination of a number of NPC proteins. (B). Both Trolox and Uric acid protect against METH-induced NPC death and decrease reactive oxygen species-induced nitrotyrosination (** p <0.01, ANOVA).

We next determined if compounds such as Trolox, a water-soluble analog of Vitamin E, and uric acid (UA), that have broad antioxidant activity could block the effects of METH on NPCs. As shown in figure 5A, both Trolox (10uM) and UA (250uM) resulted in marked decreases in nitrotyrosination of many NPC proteins. A lower dose of UA (25uM), did not decrease METH-induced oxidative stress in NPCs. Further, METH-treated NPC viability was increased by both Trolox and UA (Figure 5B), indicating that antioxidants can confer partial protection against METH-induced NPC death. Importantly, the lower dose of UA (25uM), which did not decrease nitrotyrosination, also did not protect against METH-induced AHP cell death.

### METH leads to nitrotyrosination and decreased activity of PKM2 in NPCs

To identify individual NPC proteins that may be nitrated in response to METH treatment, we immunoprecipitated METH-treated lysates with antibody to 3-nitrotyrosine. Captured proteins were eluted, trypsinized, and analyzed by mass spectrometry; the resultant peptides and their proteins were identified using commercial database searching programs. In our experiments, the additional gel separation and gel extraction steps resulted in some sample loss and low sequence coverage, which was sufficient for identification of the proteins but not the specific observation of the nitrated peptides. Seventeen proteins were identified in 3-NT immunoprecipitated METH-treated NPCs that were not observed in untreated NPCs, including pyruvate kinase M2 (Table 1).

**Table 1:** Mass spectrometric identification of proteins immunoprecipitated by antibody to 3-nitrotyrosine

Heat Shock proteins	
	grp75

	Tumor rejection antigen gp96

**Cytoskeletal proteins**	

	Tropomodulin

	Desmoplakin isoform 1,2

**Translation factors**	

	Aspartyl-tRNA synthetase

	Eukaryotic translation elongation factor 1 delta

	Ribosomal protein L6

	Acid ribosomal phosphoprotein P0

**Mitochondrial proteins**	

	mitofilin

**ER proteins**	

	ribophorin II

	calnexin

**Others**	

	Procollagen-lysine, 2 oxoglutarate 5-dioxygenase 3

	hnRNP R

	Solute carries family 3, member 2

	82-kD FMRP interacting protein

	Histone H1.2

	Pyruvate kinase M2

**Figure 6:**
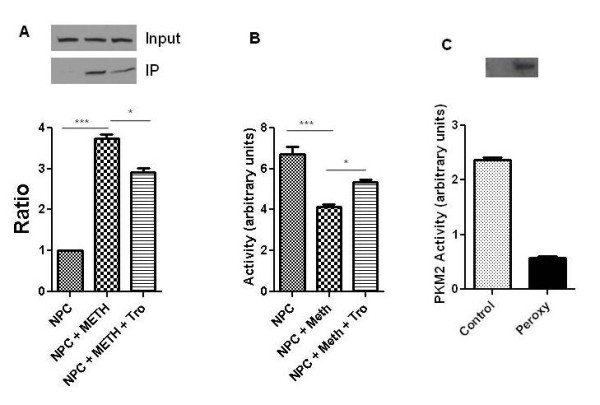
**METH induces nitrotyrosination of PKM2 and inhibits its activity**. A. NPC lysates were immunoprecipitated with antibody to 3-nitrotyrosine, and ratio of nitrotyrosinated to total PKM2 is quantified. B. Assessment of pyruvate kinase activity in NPC lysates. C. 3-nitrotyrosine Western blotting (above) and pyruvate kinase activity (below) of recombinant PKM2 (control or peroxynitrite treated). *p <0.05, ***p <0.001 ANOVA.

Since we found that METH preferentially acts on proliferating NPCs, we next sought to confirm nitrotyrosination of pyruvate kinase M2 (PKM2), a protein that mediates cell proliferation. Western blot analysis demonstrated that PKM2 is expressed in proliferating NPCs, but not in terminally differentiated cultured neurons (data not shown). We immunoprecipitated METH-treated NPC lysates with 3-NT antibody, and probed for the presence of PKM2 by Western blot (Figure 6A). METH treatment resulted in a marked increase in nitrotyrosinated PKM2, and this increase was partially blocked by Trolox. We next determined whether METH treatment affected the pyruvate kinase activity of NPC lysates. Indeed, METH treatment reduced PK activity, and this reduction was partially rescued by Trolox (Figure 6B). Thus, METH treatment of NPCs results in nitrotyrosination of PKM2 and a concomitant loss in PK activity.

We next sought to determine whether oxidative and nitrosative stress can directly inhibit activity of PKM2. Treatment with peroxynitrite resulted in nitrotyrosination of recombinant PKM2 and in diminished PK activity (Figure 6C).

### Identification of nitrotyrosinated residues of PKM2

**Figure 7:**
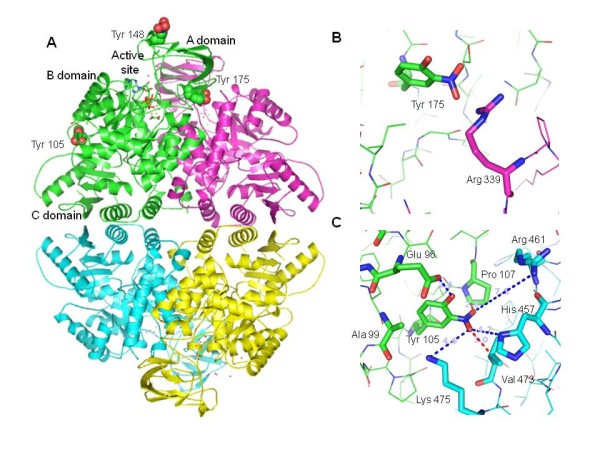
**Modeling of the nitrated tyrosine residues of PKM2**. A) Location of the nitrated tyrosines on one subunit of the PK tetramer. Nitrated tyrosine residues are represented as sticks colored by atom type (green, carbon; blue, nitrogen; red, oxygen). Detailed view of the environment of the nitrated tyrosines. B) Tyr 175 at the interface of the A-domain (green) and B-domain of neighbor subunit (magenta). C) Tyr 105 at the B/C interface. Possible and actual hydrogen bonds are represented as dashed blue lines. Steric contacts are indicated with dashed red lines. Modeling and figures were done with the program PyMOL (TM Schrodinger, LLC).

We next attempted *in *vitro studies to determine the possible amino acid sites on PKM2 that can be nitrotyrosinated. *In vitro *nitrated recombinant PKM2 was analyzed by mass spectrometry along with control protein not exposed to peroxynitrite. The sequence coverage percentages were 76% and 66%, respectively. All of the tyrosine residues aside from Y161 were detected. Control PK did not contain any nitrated residues while the in vitro nitrated PK contained three nitrotyrosine residues (Table 2). We next modeled the effects of nitrotyrosination on the known crystal structure of PKM2. PKM2 functions as a tetramer, each subunit of which is comprised of three domains (Figure 7A). Of the two nitrotyrosines located in domain A, Tyr 175 is closest to the active site cleft, residing 14.6 Å from the γ-phosphate of the ATP (Figure 7B). The third nitrated tyrosine residue, Tyr 105, is located at the interface between domains B and C (Figure 7A,C), in close proximity to the allosteric inhibitory site of the enzyme.

**Table 2:** Summary of the identification of nitrated tyrosines of PKM2 identified by mass spectrometry

Peptide sequence	Charge	Xcorr score	m/z (Da)	MH+(Da)	Delta M (ppm)
**ITLDNAY_N02_MEK**	2	2.16	621.788	1242.569	1.13

**VY_N02_VDDGLISLQVK**	2	2.16	747.394	1493.780	-2.90

**TATESFASDPILY_N02_RPVAVALDTK**	3	3.40	837.428	2510.269	-3.42

## Discussion

We found that METH decreased hippocampal NPC proliferation, increased apoptosis, and led to increased oxidative and nitrosative stress in a well-characterized *in vitro *system. Further, we identified NPC proteins that were nitrotyrosinated in response to METH, and showed that nitration of one NPC protein implicated in cell proliferation, PKM2, inhibited its activity. Thus, impairment of PKM2 and other NPC proteins via nitration may contribute to impairment of hippocampal neurogenesis in the setting of METH abuse.

It has become increasingly recognized that drugs of abuse can inhibit adult hippocampal neurogenesis, with potentially adverse consequences on regeneration and cognitive function [31,32]. Few studies, however, have focused on how METH may affect neurogenesis. In gerbils, acute METH exposure resulted in a decrease in proliferating NPCs within the hippocampal DG [39]. More recently, short-term administration of stimulant drugs, including METH, resulted in a trend toward decreased hippocampal subgranular zone NPC proliferation in rats [35]. In addition, rats that chronically or regularly self-administered METH over the course of several months also exhibited marked decreases in hippocampal neurogenesis [33]. A recent *in vitro *study also suggested that METH can impair hippocampal NPC function by inducing apoptosis [36]. Here, we demonstrate that METH inhibits adult hippocampal NPC proliferation, and at higher concentrations impairs survival without affecting neuronal differentiation. METH has recently been found to exert similar effects on proliferation and survival of subventricular zone (SVZ) NPCs [40]. In that study, however, much lower concentrations of METH inhibited neuronal differentiation in SVZ neurospheres. Taken together, these studies suggest that METH affects NPC survival, proliferation, and differentiation in a concentration-dependent manner. Importantly, the lower range of METH concentrations that impaired NPC function in our study are slightly lower than that which causes toxicity to mature neurons, and may be within the dynamic range of concentrations in the brains of tolerant METH abusers during binges [41-43]. Overall, these studies suggest that METH use can result in decreased hippocampal neurogenesis and may contribute to cognitive dysfunction.

An understanding of the cellular and molecular mechanisms by which METH affects NPCs may provide new insights into neurodegeneration and regeneration. A recent *in vitro *study found that METH, at similar concentrations to those used in this study, can act directly upon NPCs to decrease proliferation, induce oxidative stress, and result in dysregulation of the mitochondrial fission protein DRP1 and concomitant mitochondrial fragmentation [36]. Similarly, in lymphocytes, METH causes increases in intracellular calcium and disruption of the electron transport chain in mitochondria [44]. Oxidative stress has also been implicated in the impairment of hippocampal neurogenesis by alcohol, which is prevented by the synthetic antioxidant ebselen [45]. Surprisingly, in some situations oxidative stress may enhance neurogenesis. Deficiency of the antioxidant enzyme superoxide dismutase 1 (SOD1), for example, results in higher numbers of newly generated hippocampal neurons compared to wild type animals following cranial irradiation, an effect that is potentially mediated through increased oxidative stress [46]. The effects of NO and nitrosative stress on NPC function are also not fully understood [47]. Several studies suggest that NO donors decrease NPC proliferation and drive differentiation toward the neuronal lineage, while inhibitors of NOS may increase proliferation and decrease differentiation [48,49]. A decrease in transcription of the oncogene N-myc may play a role, as its levels correlate with NO-induced changes in NPC proliferation and differentiation [50]. We found striking evidence of both oxidative and nitrosative stress in NPCs upon METH exposure, and this was accompanied by decreased proliferation. Interestingly, we did not note significant effects upon NPC differentiation, nor did we find that METH affected survival of NPCs that had already begun to differentiate. Thus, METH appears to exert cell-stage specific effects, with proliferating NPCs preferentially affected as compared to differentiating cells.

A major consequence of oxidative and nitrosative stress is protein tyrosine nitration, which results from excessive peroxynitrite formation [51]. Protein tyrosine nitration is a selective process, where only a few proteins get nitrated and only a few tyrosine residues are modified within each protein The nitration of tyrosines does not predictably result in loss of function, as some proteins may exhibit either no measurable change in function or even gain in function upon nitration [51]. In a growing number of neurodegenerative disorders, however, tyrosine nitration has been implicated in disruption of protein function with adverse pathological consequences. In Parkinson's disease, tyrosine nitration can lead to increased aggregation of alpha-synuclein, potentially contributed to the increased formation of Lewy bodies [52]. Similarly, nitration of tau inhibits the ability of monomeric tau to promote microtubule assembly, increases self-aggregation, and facilitates its incorporation into neurofibrillary tangles, one of the pathological hallmarks of Alzheimer's disease [53,54]. In addition, the protein L-prostaglandin D synthase (L-PGDS) was found to be highly nitrotyrosinated in the cerebrospinal fluid of HIV-infected individuals as compared to non-HIV-infected individuals, and nitration of the protein diminished its activity [55]. Given the ability of prostaglandins to modulate a wide variety of regulatory pathways, it was hypothesized that nitration of L-PGDS may play a role in the pathogenesis of HIV dementia. Using mass spectrometry, we identified 17 proteins in the 3-NT immunoprecipitate of METH-treated NPCs. These proteins encompass diverse cellular functions, including synthesis of proteins, formation and maintenance of cytoskeletal structure, and energy production. The varied functions of the proteins we identified are consistent with the wide functional range of nitrated proteins identified in neurodegenerative disorders. Interestingly, Gene Ontology analysis indicates that the largest class of nitrated proteins found in *in vivo *disease models subserve energy metabolism, likely due to involvement of these proteins with oxidative and nitrosative-stress generating redox reactions [56].

We focused on the M2 isoform of pyruvate kinase because of its role in cellular energetics and its expression in proliferating cells. PKM2 is a glycolytic enzyme that converts phosphoenolpyruvate to pyruvate with phosphorylation of ADP to ATP. The M2 isoenzyme PKM2 is expressed in cells with high rates of nucleic acid synthesis, including tumor cells and progenitor cells, and it has been suggested to play an important role in cell proliferation [57]. Recent studies have shown that PKM2 expression is required for cancer metabolism and tumor growth [58]. Interestingly, RNAi approaches to reduce expression of PKM2 inhibit proliferation and tumor growth in a lung cancer xenograft model [59] and in glioma cells [60]. We found that nitration of PKM2 was associated with reduced pyruvate kinase activity and decreased proliferation of NPCs. Thus, post-translational modification of PKM2 by nitration lowers its activity and may be an important mechanism by which METH exerts its inhibitory effects on proliferating NPCs. Notably, nitration may be one of several post-translational modifications that affects PKM2 activity. PKM2 was recently identified as undergoing oxidative modification, as evidenced by increased protein carbonylation, in the brains of patients with mild cognitive impairment (MCI) in a proteomics approach. Interestingly, the increased oxidative modification of PKM2 was associated with reduced enzymatic activity [61].

We found that only three of the nine tyrosine residues of PKM2 were nitrated, consistent with the notion of biological specificity of nitration sites. The crystal structure of pyruvate kinase demonstrates that the three nitrated tyrosines are surface-exposed (Figure 7) [62], an apparent prerequisite to nitration. However, not all exposed tyrosine residues are capable of being nitrated. Although there is no amino acid consensus sequence that defines sites of tyrosine nitration, the presence of neighboring acidic and turn-inducing amino acids as well as the relative paucity of neighboring cysteines and methionines appears to favor nitration [63]. Examination of the sequences from -5 to +5 amino acids relative to the three nitrated tyrosines in PKM2 reveals the presence of six acidic amino acids, four turn-inducing amino acids, and only one cysteine and one methionine. Thus, the selective nitration of these three PKM2 tyrosines is consistent with previously described findings on the selectivity of protein tyrosine nitration.

Nitrotyrosination of PKM2 may affect both the active site and the allosteric site of the enzyme. The active site of PKM2 is at the cleft between domains A and B of each subunit, and the conformational flexibility of these domains results in the opening and closing of the active site cleft, thereby modulating enzymatic activity [64]. Nitration of Tyr 175 may result in hydrogen bonding to the guanidinium group of Arg 339 in a neighboring subunit (Figure 7B). This is likely to result in changes in mobility or positioning of domain A, thereby modifying enzymatic activity. Notably, a precedent for changes in domain mobility affecting PK enzymatic activity has been established [65]. Nitration of Tyr 105 may affect the allosteric site which, when bound by phenylalanine results in an inhibition of enzymatic activity [66]. At the interface between domains B and C, Tyr 105 is close to the Phe binding site identified in the PKM1 isoform. The nitro group at the benzyl 3 position of Tyr 105 may induce local distortions in a similar manner to the binding of the bulky phenyl group, thus affecting the domain B/C interface [66]. Our model of the nitrated tyrosine (Figure 7C) shows that the addition of the nitro group to Tyr 105 generates steric conflicts with Pro 107 (B domain) and Val 473 (C domain). In addition, the nitro group may form additional hydrogen bonds with Lys 475, His 457 and Arg 461 of the C domain (Figure 7C), thereby potentially altering the structure of the allosteric site.

## Conclusions

Overall, the observation that hippocampal neurogenesis is impaired by METH has several important implications. Given the emerging evidence suggesting a role for continuous hippocampal neurogenesis in cognitive functioning, a METH-induced reduction in newly generated neurons may impair the maintenance of hippocampal-dependent learning and memory [19,67,68]. Indeed, spatial working memory, a task dependent upon hippocampal integrity, is impaired by METH [69]. In addition to countering the deleterious effects of METH on mature neuronal cells, therapeutic strategies may need to be directed at increasing and optimizing neurogenesis in this patient population. More importantly, however, our studies point to a role for METH-induced oxidative and nitrosative stress in impacting NPC proliferation and survival. A better understanding of the effects of nitrotyrosination on NPC protein function will likely yield insights into the pathogenesis of neurodegeneration in the setting of METH abuse and other CNS disorders in which oxidative and nitrosative stress play important roles.

## Materials and methods

### Cell culture

The adult hippocampal NPCs used in this study were clonal stem cells derived from the hippocampus of adult Fischer 344 rats, and have been previously shown to fulfill the characteristics of multipotent neural precursor cells both *in vitro *and *in vivo *[70]. NPCs were propagated in proliferation media (DMEM/F12 medium containing N2 supplement, L-glutamine [2mM], and FGF-2 [20 ng/mL]), as previously described [70]. For differentiation, NPCs were trypsinized and plated in DMEM/F12 containing N2 supplement, L-glutamine (2mM), fetal bovine serum (FBS) (1%; v/v), and retinoic acid (1 μM) for up to four days. For most experiments, NPCs were plated directly on laminin-coated 24-well plates to minimize detachment of cells during toxicity assays. Initial plating density was 25,000 cells/cm^2^. Cells from passage numbers 15 through 21 were used in all experiments.

### Immunocytochemistry and quantification of cell types

NPCs were fixed with 4% (w/v) paraformaldehyde (PFA) and washed with Tris buffered saline (TBS), prior to incubation in blocking solution (TBS with0.25% (v/v) Triton-X and 0.5% (v/v) FBS). Primary antibodies were diluted in blocking solution as follows: anti-bromodeoxyuridine (BrdU) (1:400, rat; Accurate, City, State), anti-nestin (1:1,000, mouse; Sigma, St Louis, MS), anti-β-tubulin (1:1,000, mouse, BAVCO, City, State), anti-RIP (give full spelling) (1:25, mouse, Hybridoma Bank, City, State), anti-glial fibrillary acidic protein (GFAP) (1:1,000, rabbit, Dako, City, State), and incubated with NPCs overnight at 4°C. The detection of BrdU required treatment of cells with 1M HCl at 37°C for 30 min prior to application of the primary antibody. After washing twice with TBS, NPCs were incubated with the appropriate Alexa-fluor conjugated secondary antisera (1:250, Invitrogen), followed by washing and counterstaining with DAPI to label all nuclei. Positive cells were quantified in at least 10 randomly selected fields (20X magnification) in each of at least three wells per condition using a fluorescent microscope, and each experiment was performed in triplicate.

### Assays of cell viability and apoptosis

NPCs maintained under proliferative conditions were exposed to METH (+), which was obtained from the National Institute of Drug Abuse. Cell viability was assessed by exclusion of either trypan blue or 7-amino actinomycin D (7-AAD). Trypan blue was added to a final concentration of 0.2% (w/v) to treated cells, followed by direct visualization of cells under a light microscope (give magnification). The percentage of dead cells with blue nuclei were quantified in at least 10 randomly selected fields in each of at least three wells per condition. Alternatively, treated cells were trypsinized, resuspended in phosphate buffered saline, pH = 7.4 (PBS), and incubated with 7-AAD (1 ug/mL, Calbiochem, City, State) for 15 minutes, followed by flow cytometric analysis (Becton-Dickinson, City State). The percentage of cells that stained with 7-AAD (dead cells) was quantified in at least three separate experiments.

To assess for apoptosis, cells were fixed, washed, and terminal deoxynucleotide nick end labeling (TUNEL) staining was performed using the Promega DeadEnd Fluorescent TUNEL Labeling Kit according to manufacturer's instructions. Cells that had undergone apoptotic DNA fragmentation incorporated FITC-labeled dUTP. TUNEL positive cells were detected by fluorescence microscopy (give magnification).

### Detection and inhibition of oxidative stress in NPCs

Oxidative and nitrosative stress-induction was measured by several methods. Carboxy H2 DCFDA (10 uM, Invitrogen Molecular Probes) is an indicator compound that passively diffuses into cells and is trapped inside upon cleavage by intracellular esterases. Upon interaction with reactive oxygen species, the fluorescent product DCF is formed. NPCs maintained under proliferative conditions were treated with several concentrations of METH for varying time points, followed by incubation with Carboxy H2DCFDA at 37°C for 30 minutes in a 5% (v/v) CO_2 _incubator. DCF formation was visualized by fluorescence microscopy (give magnification). Nitrite production was measured from the supernatants of METH-treated NPCs using the Promega Griess Reagent System (Cat # 2930) as per the manufacturer's directions.

Alternatively, the formation of nitrotyrosination adducts on proteins, which represents a common end-product of several oxidative and nitrosative stress pathways [38] was assessed. NPCs were incubated with either METH or three potent inducers of oxidative stress, staurosporine (0.2 μM, Sigma), 3-nitropropionic acid (1 mM, Sigma), or 2,3-dimethoxy-1,4 naphthoquinone (1 mM, Sigma). Cell lysates were collected at varying time points and subjected to either slot-blot analysis (BioRad) or Western blot analysis with antibody to 3-nitrotyrosine (1:1000, Upstate, Charlottesville, VA) and beta-actin (1:5000, Sigma). Blots were developed using the ECL Plus kit (Amersham) followed by quantitative densitometry (Image J, NIH). Each experiment was conducted at least three separate times.

To determine whether antioxidants protect against METH-induced NPC death, NPCs were preincubated for 2 hrs with either Trolox or Uric acid, followed by addition of METH (250 μM). Cell lysates were analyzed by Western blot 48 hours later. The concentrations of Trolox (10 μM) and uric acid (25 μM and 250 μM) used were typical of that employed in the literature; of note, levels of uric acid present in the serum of normal humans range from 250 μM to 350 μM [71,72].

### Mass spectrometric analysis

NPC cell lysate was mixed with 3 µg of monoclonal anti-nitrotyrosine antibody (Millipore #05-233) in an end-over-end mixer for 1 hour at room temperature. The mixture was then added to Protein G beads that had been pre-washed with PBS, and mixed for 1 hour at room temperature. The beads were then centrifuged and washed extensively, and the protein bound to the beads was eluted with 25ul of 1x SDS sample buffer. Eluted proteins were separated on NuPage 4-12% Bio-Tris gels( Invitrogen NP0335) and stained with the SilverQuest Staining kit (Invitrogen #LC6070). Bands were cut and sliced into 1 × 1 mm pieces; then rinsed with methanol and ammonia biocarbonate. After incubation with Trypsin at 37C overnight, the tryptic peptides were extracted and the supernatant was collected for LCMS-MS analysis using an LTQ Orbitrap (Thermo Fisher). A C18 column (75um id, YMC ODS-AQ 5um particles with 120A pore size) was used in 2D nanoLC with gradient (5-60% of 0.1% Fomic acid/90% acetonitrile) over 30 minutes with a flow rate of 300nL/min. Data-dependent MS/MS mode was applied and the resulting MS/MS spectra were analyzed using Mascot (Matrix Science, London, UK; version Mascot) and X! Tandem (The GPM, thegpm.org; version 2007.01.01.1). They were set up to search the NCBInr (selected for Rodentia, version 2007.10.15, 137641 entries) with dynamic modification oxidation of Met (+15.995 Da) (peptide and MS/MS tolerance 0.1 and 0.8 Da).

In vitro nitrated rabbit pyruvate kinase and control rabbit pyruvate kinase were desalted with desalting spin columns (Pierce), dried using a speed vacuum instrument, resuspended in 100 mM ammonium bicarbonate and digested with trypsin (1:20, w/w). The peptides were resuspended in 0.1% formic acid and separated by *on-line *reversed-phase nanoscale capillary liquid chromatography (Eksigent, Dublin, CA) over a 120 min gradient (Solvent A, 0.1% formic acid, solvent B 0.1% formic acid in 90% acetonitrile) followed by analysis by ESI-tandem mass spectrometry using a LTQ-Orbitrap mass spectrometer (Thermo Fisher, San Jose, CA). The tandem mass spectra of rabbit pyruvate kinase sample set were extracted and initially analyzed by Thermo Proteome Discoverer 1.1. MS/MS spectra were analyzed using Sequest (Thermo Fisher Scientific, San Jose, CA, USA; version 1.1.0.263) and X! Tandem (The GPM, thegpm.org; version 2007.01.01.1). The rabbit pyruvate kinase database (1 entry) was searched with the following parameters: enzyme, trypsin; precursor ion mass tolerance, 0.100 Da; fragment ion mass tolerance, 0.800 Da; maximum missed cleavage sites, 2; and dynamic modifications, oxidation of Met (+15.995 Da) and nitration of Tyr and Trp (44.985 Da). The nitrated peptide hits were manually validated. Scaffold (version Scaffold_3_00_03, Proteome Software Inc., Portland, OR) was used to validate all MS/MS based peptide and protein identifications. Peptide identifications were accepted if they could be established at greater than 95.0% probability as specified by the Peptide Prophet algorithm [73]Protein identifications were accepted if they could be established at greater than 99.0% probability and contained at least 2 identified peptides. Protein probabilities were assigned by the Protein Prophet algorithm [74].

### Nitrotyrosination and activity of native PKM2 protein

Peroxynitrite (Cayman Chemicals) was used for nitration of rabbit PKM2 native protein (GenWay Biotech). 89 μg of PKM2, 10 μl of 10mM NaHCO3 and 1 μl of OONO^- ^were placed in an eppendorf tube for 10 min at RT, followed by addition of beta mercaptoethanol to terminate the reaciton. Samples were then analyzed by western blot.. Two sets of samples were incubated separately with Anti-Nitrotyrosine (3-NT; Cat # 16-163, Millipore) primary antibody (1:500) followed by horseradish peroxidase-conjugated Anti Mouse (1:5000), and with PKM2 primary antibody (1:1000) followed by horseradish peroxidase-conjugated Anti-Rabbit Secondary Antibody (1:5000), and both were detected using ECL Plus.

The Bio Vision Pyruvate Assay Kit (Cat # K709-100) was used to measure the activity of pyruvate kinase as per manufacturer's instructions.

## Competing interests

The authors declare that they have no competing interests.

## Authors' contributions

AV, LU, ZC, LR, CA, and ML carried out the experiments. MAB performed the structural modeling. AV, RC, HS, and AN drafted the manuscript. All authors read and approved the final manuscript.
